# Relapsing Polychondritis

**DOI:** 10.1155/2014/791951

**Published:** 2014-09-30

**Authors:** Beata Sosada, Katarzyna Loza, Ewelina Bialo-Wojcicka

**Affiliations:** Department of Dermatology, Miedzyleski Specialist Hospital in Warsaw, ul. Bursztynowa 2, 04-479 Warsaw, Poland

## Abstract

Relapsing polychondritis (RP) is a rare systemic disease characterized by recurrent, widespread chondritis of the auricular, nasal, and tracheal cartilages. Additional clinical features include audiovestibular dysfunction, ocular inflammation, vasculitis, myocarditis, and nonerosive arthritis. Although the cause remains unknown, the etiology is suspected to be autoimmune. We describe a case of a 31-year-old woman with a four-month history of bilateral auricular and nasal chondritis. Infectious and neoplastic diseases were excluded by imaging and laboratory examinations. RP was diagnosed based on three McAdam's criteria. The patient was medicated with oral prednisolone and methotrexate with positive clinical response. In this case clinical history and detailed physical examination were fundamental in concluding the correct diagnosis and administrating the appropriate medication.

## 1. Introduction

Relapsing polychondritis (RP) is a rare inflammatory disease primarily affecting the cartilaginous structures of the ear, nose, joints, tracheobronchial tree, and cardiovascular system. Cardiovascular and respiratory complications of RP are associated with high morbidity and mortality. The first case of RP was described in 1923 by Jaksch-Wartenhorst [1]. The term “relapsing polychondritis” was first used by Pearson et al. in 1960 in their review of 12 cases [2]. RP was usually observed in the fourth and fifth decade of life with no sex predilection [3–5].

The McAdam's criteria were the initial diagnostic criteria of RP [3] and required meeting three out of six of the following: bilateral auricular chondritis, nonerosive seronegative inflammatory arthritis, nasal chondritis, ocular inflammation, respiratory tract chondritis, and audiovestibular damage. Modified criteria have been proposed by Damiani and Levine [4] which include meeting one McAdam's criterion plus histopathological confirmation or two McAdam's criteria plus response to corticosteroids or dapsone. Currently, the diagnosis of RP relies mostly on the criteria established by Michet et al. [5] which require the presence of a proven inflammation in at least two of three of the auricular, nasal, or laryngotracheal cartilages or the proven inflammation in one of these cartilages plus two other signs, including ocular inflammation, vestibular dysfunction, seronegative inflammatory arthritis, or hearing loss (Table 1).

The exact cause of RP is still unknown but the disease is mostly seen as an immune-mediated disease, as there is a well-documented overlap of RP with other rheumatic and autoimmune diseases [3, 6]. Although a large number of cases have been reported recently and the knowledge on the clinical spectrum, pathogenesis, and management in RP has grown considerably, only limited microscopic data is available in the literature [4, 7]. The histologic features of the chondritis include loss of basophilic staining of the cartilage matrix followed by cartilage destruction with replacement by fibrous tissue and cellular infiltration with plasma cells and lymphocytes. A rare disease RP is described occurring extremely rarely in young women.

## 2. Case Report

**Figure 1:**
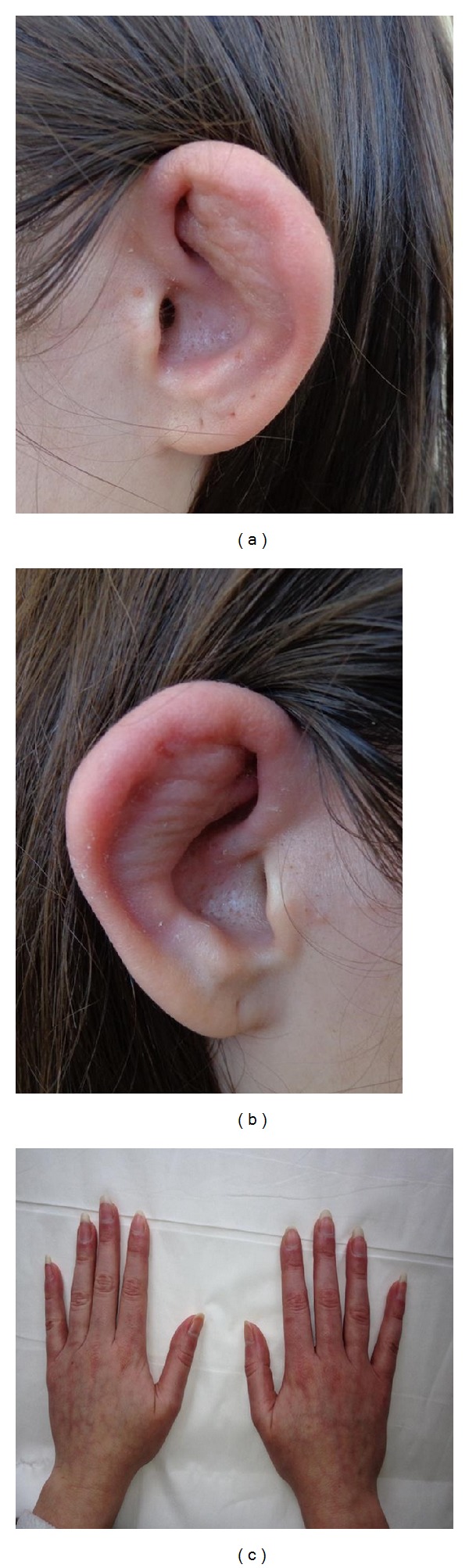
(a, b) Cauliflower ears. Swelling and erythema of the cartilaginous part of the ear, sparing the lobule which lacks cartilage. (c) The Raynaud's phenomenon.

A 31-year-old Caucasian woman was consulted in our department for recurrent swellings of both pinnae which had been present for approximately 4 months. About two weeks before coming to hospital she suffered from pain and tenderness of both auricles, the nose as well as the left elbow. Her personal and family history was unremarkable. She was a smoker (10 pack-years). During physical examination both pinnae lost their firmness, became soft and floppy, and had a cauliflower-like appearance (Figures 1(a) and 1(b)). In addition Raynaud's phenomenon was found (Figure 1(c)) and evidenced by nailfold capillaroscopy.

**Figure 2:**
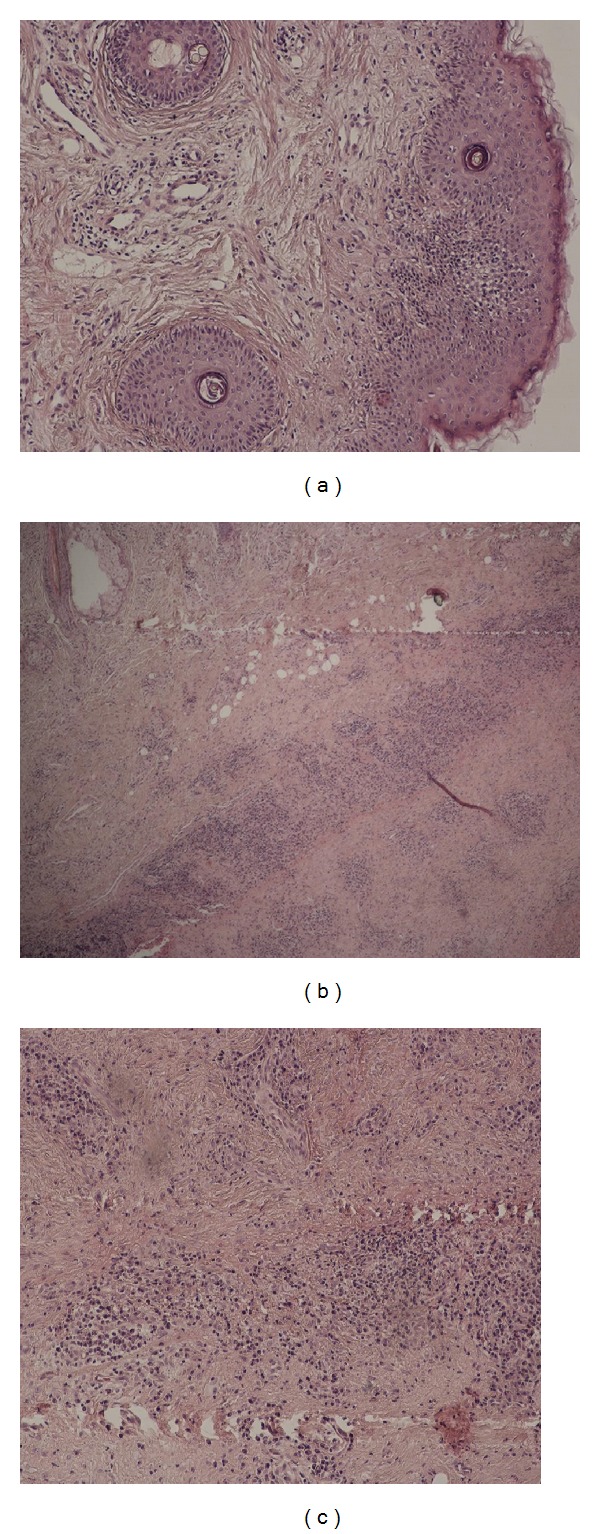
(a) The dermis contains a mild focal lymphohistiocytic infiltrate. H&E, ×100. (b) Degenerative and inflammatory changes affecting the marginal chondrocytes with loss of basophilia and poor alcian blue staining of the cartilaginous tissue. H&E, ×40. (c) The inflammatory cells infiltrate, including lymphocytes, plasma cells, and histiocytes, infiltrate the degenerative cartilage. H&E, ×100.

Routine blood investigations revealed normocytic normochromic anemia, elevated erythrocyte sedimentation rate. The rheumatoid factor was within normal limits. Other clinical parameters (urinalysis, thyroid tests, and liver function tests) resulted within normal range. Antinuclear antibodies (ANA) titer was 1 : 320. Antiphospholipid antibodies, antineutrophil cytoplasmic antibodies (ANCAs), anti-Borrelia burgdorferi antibodies IgG/IgM, rheumatoid factor, anti-HIV-1, anti-HIV-2, and VDRL tests were negative. Two biopsy specimens were taken, one from the skin and another from the cartilage of the pinna for histopathological study. Histologic pictures show cellular infiltrates by lymphocytes, neutrophils, and plasma cells, most evident in the cartilage-skin interface, as well as the reduced number of chondrocytes seen in areas of cartilage destruction (Figures 2(a), 2(b), and 2(c)).

The skin tissue was also processed for direct immunofluorescence (DIF) studies where isolated IgG staining at the BMZ was observed. Spirometry, computer tomography, and radiography of the chest did not reveal any laryngotracheobronchial symptoms. No ocular disorders in ophthalmologic consultations were found. Doppler echocardiography and electrocardiography did not reveal any abnormalities. The patient started on prednisolone 30 mg daily with improvement in symptoms. Approximately 8 weeks following discharge, while tapering prednisolone to 15 mg daily, she had recurrence of nasal pain and auricular swelling. Prednisolone dose was increased to 30 mg daily and a combination therapy with methotrexate 15 mg weekly was recommended. After 6 months, corticosteroids were reduced to 5 mg daily and methotrexate was increased to 17,5 mg weekly. The patient is still on followup with no progression during this period. Moreover, ANA titer decreased to 1 : 160.

## 3. Discussion

RP is an autoimmune disease in which target antigens are still unknown. Both circulating antibodies and immune complex deposits in the affected cartilaginous tissue could be present. Studies [8, 9] have shown that 33% of patients with RP had circulating antibodies of type II collagen in the active phase of the disease and their titres also corresponded to the disease activity. Autoimmunity to collagen type II has also been described in systemic lupus erythematosus (SLE) and rheumatoid arthritis. Other studies [10, 11] showed that the antibodies are generated against not only native and denatured collagen type II but also collagen types IX and XI, which form the major extracellular scaffold in the cartilage. Matrilin-1 is a cartilage-specific protein and is highly expressed in tracheal and nasal but not in normal adult articular cartilage [12]. Saxne and Heinegard in their studies [12, 13] revealed that an increased serum level of matrilin-1 could be found in patients with RP in the active phase, suggesting that the release of matrilin-1 resulted from the destruction of the involved cartilage. However, neither anti-collagen type II nor anti-matrilin-1 antibodies are sensitive and specific enough and consequently cannot be used for diagnostic purposes. The diagnosis of RP is largely based on the clinical features and the role of laboratory and imaging investigations is purely supportive to rule out other related or associated systemic diseases. Clinical, histopathological, and DIF features together or in combination are helpful in the final diagnosis. The treatment of RP is symptomatic and should be tailored to each individual patient based on disease activity and severity.

Glucocorticoid therapy is fundamental in the treatment of RP and is used chronically in most patients. Less severe symptoms are generally treated with nonsteroid anti-inflammatory drugs. Dapsone may also be used as an initial therapy but results in many adverse reactions. Severe symptoms of disease, including ocular or laryngotracheal involvement, systemic vasculitis, and severe polychondritis require systemic corticosteroids. In patients intolerant to, rarely unresponsive to, steroid therapy or in whom a steroid sparing therapy is required, immunosuppressants play a role. Immunosuppressive agents like methotrexate, azathioprine, and cyclosporine may be given to patients with severe respiratory or vascular involvement and to those with steroid-resistant or steroid-dependent disease. Trentham and Le [14] observed that methotrexate in dose of 17,5 mg/week was the most effective nonsteroid drug in causing symptomatic benefit and reducing the steroid requirement. Intravenous cyclophosphamide and plasmapheresis could be used in patients with organ-threatening and life-threatening diseases, including glomerulonephritis or acute airways obstruction. The autoimmune theory of pathogenesis of RP makes immunomodulatory agents (biologics) an important treatment alternative to other medical therapies. However, data from clinical trials is scarce; there are many case reports of satisfactory response to biologic therapy in RP. Standard management cannot be established due to its rarity.

## Figures and Tables

**Table 1:** Diagnostic criteria for RP.

McAdam et al. [3]	(1) Recurrent chondritis of both auricles (2) Nonerosive inflammatory polyarthritis (3) Chondritis of nasal cartilages (4) Inflammation of ocular structures (5) Chondritis of respiratory tract (6) Cochlear and/or vestibular damage (requirement—three out of six criteria)

Damiani and Levine [4]	(1) Three out of six McAdam et al.'s [3] criteria (2) One out of six McAdam et al.'s [3] criteria and a positive histologic confirmation (3) Two out of six McAdam et al.'s criteria and response to corticosteroid or dapsone (requirement—any of these)

Michet et al. [5]	(1) Proven inflammation in two out of three cartilages: auricular, nasal, and laryngotracheal (2) Proven inflammation in one of the above and meeting two other signs from ocular inflammation, hearing loss, vestibular dysfunction, or seronegative inflammatory arthritis (requirement—any of these)
